# The role of sound stimulation in production of plant secondary metabolites

**DOI:** 10.1007/s13659-023-00409-9

**Published:** 2023-10-17

**Authors:** Li Wu, Ning Yang, Meng Guo, Didi Zhang, Reza A. Ghiladi, Hasan Bayram, Jun Wang

**Affiliations:** 1 Department of Music, South-Central Minzu University, Wuhan, Hubei China; 2 Cooperative Innovation Center of Industrial Fermentation, Ministry of Education & Hubei Province, Hubei University of Technology, Wuhan, Hubei China; 3 Department of Chemistry, North Carolina State University, Raleigh, NC USA; 4 Department of Pulmonary Medicine, Koç University Hospital, Koç University, Istanbul, Turkey

**Keywords:** Sound, Music, Plant secondary metabolites, Flavonoid, Antioxidant, Immunity

## Abstract

Sound vibration is one of natural stimuli trigging physiological changes in plants. Recent studies showed that sound waves stimulated production of a variety of plant secondary metabolites, including flavonoids, in order to enhance seed germination, flowering, growth or defense. In this review, we examine the potential role of sound stimulation on the biosynthesis of secondary metabolites and the followed cascade of physiological changes in plants, from the perspective of transcriptional regulation and epigenetic regulation for the first time. A systematic summary showed that a wide range of factors may regulate the production of secondary metabolites, including plant species, growth stage, sound types, sound frequency, sound intensity level and exposure time, etc. Biochemical and physiological changes due to sound stimulation were thoroughly summarized as well, for secondary metabolites can also act as a free radical scavenger, or a hormone signaling molecule. We also discussed the limits of previous studies, and the future application of sound waves in biosynthesis of plant secondary metabolites.

## Introduction

Plants are sensitive to a wide range of environmental stresses, including salinity, heavy metals, drought, moisture, water pressure, temperature, light and sound. These environmental factors can influence plants at molecular, biochemical and physiological levels [1]. Even though plants don’t have specialized sensory organs, they are excellent at detecting external environment, including sound [2]. Sound vibrations are mechanical stimuli, which are characterized by their wavelength (Hertz, Hz), intensity (decibel, dB), speed and direction [3, 4]. According to human perception, sound is classified into three categories: sonic or audible or ordinary in the range of 20 Hz-20 kHz, infrasonic (< 20 Hz) and ultrasonic (> 20 kHz). There are three ways plants perceive sound: direct touch or vibration, soil- or water-borne medium, air-borne medium [5, 6].

Sound perception is a big advantage for plants, for sound waves are presence in nature, travel fast, and are important information sources. The potential advantages include seed germination, pollination, biotic or abiotic threats, overcoming environmental challenges, and inter-communication between plants [7, 8]. A few recent studies showed that physiological changes may be induced by sound stimulation, including gene expression, epigenetic modification, hormone signaling, seed germination, growth, flowering, defense and disease, etc. [9]. Among these changes, production of plant secondary metabolites has caught increasing attention lately [7, 10–17].

Plants produce a wide diversity of organic compounds, including primary- and secondary- metabolites [18]. Secondary metabolites are generally not essential for growth or life of plants, which are metabolic intermediates or products regulating the interaction between plants and environment. In fact, secondary metabolites play an important role of plant defense against pathogens and environmental stresses, which in turn regulate synthesis and accumulation of secondary metabolites [19, 20]. The general environmental factors include, but not limited to salinity, water, light, temperature, soil moisture, soil fertility, and chemicals, etc. They can influence plant growth and development, which are traced back to changes of secondary metabolites and phytochemical profiles [21–24]. Many secondary metabolites exhibit diverse biological and pharmacological properties [25], and therefore have been applied for disease therapy. Flavonoids are one type of abundant secondary metabolites with different biological functions in plants, which may act as a free radical scavenger [26], a chelation compound for metals [27], or a regulator for hormone signaling [28–30].

In the past two decades, the effect of sound vibrations was investigated in production of plant second metabolites [7, 31]. However, a comprehensive review in this field is still missing. In this paper, we summarized recent research of sound stimulation on biosynthesis of secondary metabolites in plants, which covered the general role of sound in transcriptional-, epigenetic- and hormone signaling-changes of secondary metabolites. It would provide fresh insights and help us better understand the role and application of plant acoustics.

## Role of sound vibration in plant physiology

### The general features of sound

**Fig. 1:**
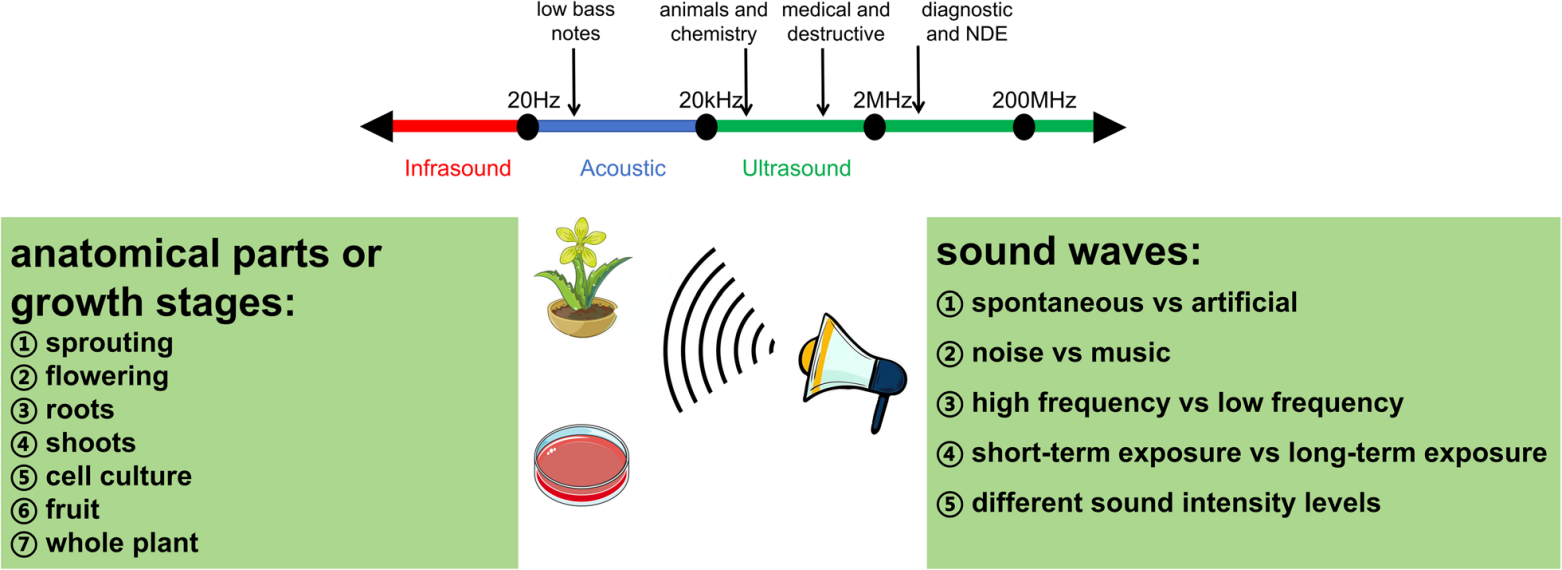
Schematic description of the exposure of plant or plant cell culture to sound waves

Sound is a mechanic vibration propagating through a gas, liquid or solid medium. It is transmitted as longitudinal waves in air or water, with particles oscillating along the direction of propagation. In solid, sound travels as longitudinal and transverse waves, with particles oscillating at right angle to the direction of propagation. Frequency (Hz), intensity (dB) and timbre are three major properties of sound, which influence sound propagation. Density and pressure, motion and viscosity of the medium all affect how sound waves travel, which makes sound propagation different in air, water and solid [32]. Plants have different parts distributed in air, liquid and solid, which makes the measurement of their response to sound complicated and interesting (Fig. 1), which is also a not-well-understood field.

When the effect of sound is investigated, a wide range of factors should be put into consideration (Fig. 1), including but not limited to: spontaneous or artificial, for example, music is artificial; noise or music; high frequency or low frequency; short-term exposure or long-term exposure; levels of sound intensity. Also, a clear description of the parts and growth stages of plant is also needed, such as sprouting, flowering, roots, shoots, cell culture, fruit or the whole plant.

### Sound perception in plants

Human can only hear audio frequency in the range of 20–20 kHz, with relatively low sound intensity (∠70 dB) to feel comfortable. So far, there is not much known about sound perception in plants, though sound emission from plants were reported to be 50–120 Hz and 20–100 kHz [33]. Increasing number of evidences indicated a variety of biological significances of sound in plants [6]. For example, plants “heard” the noise of underground water flowing and directed its root growth to reach out water sources [6, 34]. Plants “heard” the move of pollinator via flowers and responded with sweetening the nectar [35, 36], which might be a co-evolution strategy between pollinators and flowering plants for mutual benefits. Plants “heard” the noise produced by herbivores (such as insects’ chewing) and increased its defense via more production of defensive molecules [35, 36], which had been observed among pepper, tomato and cucumber. Plants “heard” sound caused by abiotic stresses such as adverse climate (such as drought, heat) and increased the expression of related genes and proteins to enhance their survival and growth [37]. Additionally, sound may also boost growth and yield [38], delay ripening [14], and facilitate post-harvesting management [14].

. The phenotypes induced by sound stimulation include seed germination, plant growth, crop production, immunity defense, resistance to harsh environment [38]. How plants initiated these physiological changes could be traced down to changes at molecular and biochemical levels. Sound stimulation regulates gene transcriptions, epigenetic modification, protein activity, hormone signaling and metabolite levels [11, 39–43]. The immune activation is usually linked to enhanced production of secondary metabolites in plants.

## Role of sound vibration in biosynthesis of plant secondary metabolites

**Fig. 2:**
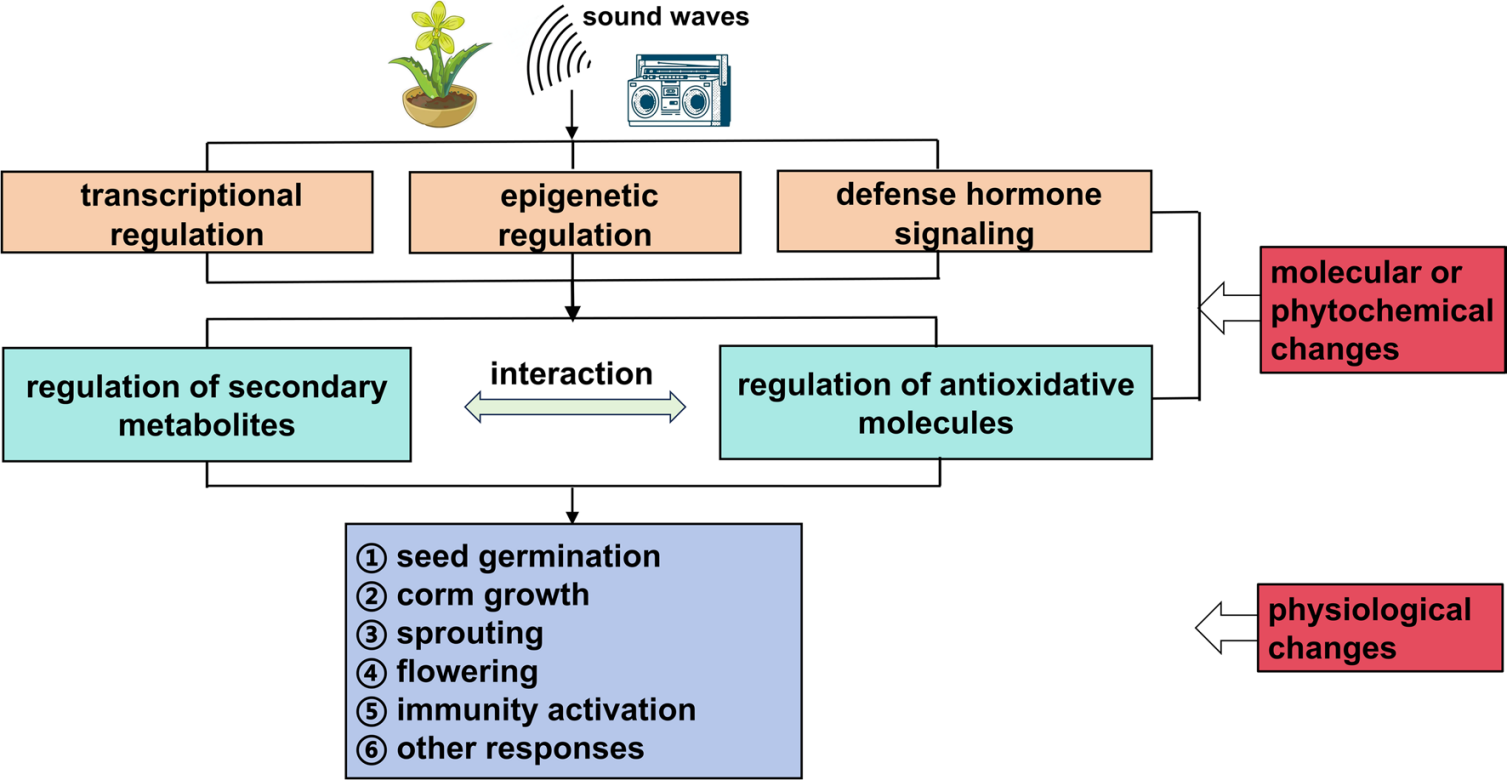
Potential sound stimulation pathways for biosynthesis of plant secondary metabolites

The physiological changes induced by acoustic waves can be traced down to changes at molecular and phytochemical levels. As shown in Fig. 2, sound waves stimulate transcriptional-, epigenetic- and hormone signaling-changes, which lead to the next-step modulation of secondary metabolites or antioxidants. The later may result in phenotypes for seed germination, corm growth, sprouting, flowering, immunity activation or others. Besides, there exist interactions between secondary metabolites and antioxidants.

### Transcriptional changes

In 2015, Kim et al. from Korea reported that vibration treatment at 1 kHz for tomatoes reduced ethylene production in their fruits, as well as procrastination of fruit ripening [14]. Analysis by real-time fluorescence quantitative real-time PCR (qRT-PCR) showed that ethylene biosynthesis-related genes (ACS2, ACS4, ACO1, E4, E8) and maturation-regulated genes (RIN, TAGL1, and NOR) were significantly reduced in the presence of sound vibration treatment. One year later, Kim et al. published another study about whether RIN and HB-1 directly activates the transcription of 1-aminocyclopropane-1-carboxylic acid (ACC) synthase (ACS) and ACC oxidase (ACO) or not. Results from transcriptional activation analysis in Arabidopsis thaliana leaf protoplasts and transient analyses of Nicotiana tabacum showed that RIN might regulate ethylene biosynthesis-related genes via binding to CArG-boxes, therefore suppressing fruit ripening in tomatoes [44].

In 2019, effects of sound waves at a variety of frequency (250 Hz to 1.5 kHz) were investigated on flavonoids in alfalfa, cauliflower, kale and carrot [13]. Joo Yeol Kim and co-workers reported that a few factors, including growth stage, species, frequency and sonic exposure time, influenced total flavonoid contents. Sound exposure improved yields of flavonoids, with 200% for alfalfa (250 Hz), 35% for broccoli sprouts (800 Hz) and 85% for carrot sprouts (1 kHz), respectively. Additionally, genetic expression of flavonoid biosynthesis pathway was positively correlated with production of flavonoid, which included general phenylpropanoid pathway-related genes, flavonoid biosynthesis-related genes and other related genes.

In 2023, Azgomi et al. from Iran investigated the effect of music and noise on savory. All acoustic treatments stimulated germination, growth, and biomass accumulation. The treatments increased the activity of the phenylalanine ammonia-lyase (PAL) enzyme and total phenolic concentrations. The highest concentration of flavonoids was positively correlated with a sharp up-regulation of the basic leucine zipper transcription factor gene [7]. In addition, the expression of α-terpineol synthase gene, secondary metabolites, and growth enhancement were positively correlated. Though only few studies had been performed so far, results already proved the connection among genetic adaption, fruit ripening, growth and acoustic waves.

### Epigenetic changes

Besides genetic regulation, Joo Yeol Kim and collaborators also investigated epigenetic changes caused by 1 kHz sound (6 h treatment) [14]. Western blotting was applied for measurement of histone modifications in tomato fruit. Through regulation of histone modifications enzyme genes, sonic vibration inhibited the transcription factors (RIN, NOR, and TAGL1). Additionally, results of ChIP assays showed that histone-modifying enzymes do not directly bind to the promoters of ethylene biosynthesis genes. Thus, sound-induced epigenetic modification (histone methylation or acetylation) was considered responsible for delayed fruit ripening in tomatoes. One of the key epigenetic gene regulation strategies is histone modification-mediated chromatin remodeling, which triggers defense priming [45].

In 2020, Jihye Jung and collaborators from Korea investigated how sound waves mediated epigenetic modifications in immune activation in Arabidopsis thaliana against the root pathogen *Ralstonia solanacearum*, with combined techniques of chromatin immunoprecipitation (ChIP) and microRNA sequencing [46]. Through exposure to 10 kHz sound, a number of H3K27me3 modification occurred in the promoter regions of aliphatic glucosinolate biosynthesis and cytokinin signaling genes, which caused transcriptional changes to enhance immunity. Jung et al. thoroughly studies all biosynthetic genes of glucosinolate. They found that the H3K27me3 modification suppressed the expression of glucosinolate biosynthetic genes and triggered induced resistance in plants, which was alleviated when the upstream genes of glucosinolate pathways were disrupted. Further evidences showed that sonic vibration induced the priming of glucosinolate-related genes in Arabidopsis, which activated resistance against *R. solanacearum*.

Sonic treatments can trigger plants to produce a large number of histone modifications, thus enhancing genetic expression of secondary metabolite (such as glucosinolates), improving immunity in organism and promoting antimicrobial effects. Acoustic stimulation can also increase disease resistance in plant cells.

### Biochemical and physiological changes

In 2003, one acoustic study by Qin et al. from China was carried out on Chinese cabbage and cucumber at two growth stages (seedlings, mature plants), with results showing that sound exposure caused significantly higher production of polyamines and more O_2_ uptake compared to the control and thus enhanced plant growth rates [11]. In the following year, another Chinese researcher Wang et al. reported changes of indole-3-acetic acid (IAA) and abscisic acid (ABA) upon sound exposure [12]. The results from indirect enzyme-linked immunosorbent assays showed that the concentration of IAA increased and that of ABA decreased, in accordance with better healing of plant tissues and faster differentiation of mature healing tissues.

In 2004, the effects of pulsed electric field (PEF) were explored by Ye et al. from China on the growth and secondary metabolite production of plant cell culture (yew suspension culture) [10]. Exposure to PEF for 30 min significantly increased the production of intracellular bioactive secondary metabolite Taxuyunnanine C, reactive oxygen species (ROS), and phenolics, which were defensive responses in plant cells.

In 2014, Cai et al. from Korea investigated the effect of audible sound on germination and growth of mung beans in the lab [47]. The results showed that sound stimulation shortened the germination period of mung beans, with different effects from varied frequencies.

Later on, Choi et al. from Mexico studied the effect of sound on the resistance of Arabidopsis thaliana against Botrytis cinerea infection in 2017 [48]. Results from the microarray and qRT-PCR analysis showed that the exposure at 1000 Hz with 100 decibels led to up-regulation of a few defenses and SA-responsive and/or signaling genes in the infected Arabidopsis plants. The level of salicylic acid increased in the sound-stimulated plants after pathogen inoculation. Choi and collaborators proposed that sonic treatment activated plant defense, which in turn enhanced sound-induced resistance in Arabidopsis against B. cinerea.

Similar results were reported in strawberry studies. In 2018, Ozkurt et al. from Turkey treated strawberry plants at 1000 Hz with varies intensity levels (95, 100, and 105 dB). A wide range of growth parameters were measured for fresh and dry weights of the roots and above-ground parts of the strawberry plants, including concentrations of vitamin C, total sugar, total acid and total phenol contents [17], which indicated that the sound wave at 1000 Hz influenced plant growth and fruit quality.

In 2021, Razavizadeh et al. from Iran investigated the effect of emitted sound from an aeroponic culture on saffron [16]. The sound frequency was in a wide range, the sound intensity was at 77 dB, and high-performance liquid chromatography was applied to measure crocin, picrocrocin, and safranal in the stigmas. Levels of crocin, picrocrocin, and safranal were lowest at 1 kHz, and highest at 2 kHz. In addition, their contents during the flowering period were significantly enhanced when sounds waves were set at higher frequency (4, 8, 12, and 16 kHz).

In literature, sound regulated Ca^2+^ and ROS signaling pathways to modulate plant cells [31]. Jung et al. reported that Ca^2+^ ions in-fluxed cytosol from outside plant membrane via exposure to 1 kHz sonic wave. It indicated that Ca^2+^ and ROS species may also serve as messengers against environmental stresses, such as microbial pathogens [49].

Through stimulation of secondary metabolite biosynthesis or increase of oxygen uptake in plant cells, sound waves at appropriate intensity levels promote plant growth. These physiological changes may include shorter germination period of seeds, faster differentiation of healing tissues, and better growth of seedlings or mature plants.

## Ultrasound in biosynthesis of plants secondary metabolites

Different from ordinary sound, ultrasound or ultrasonication travels with frequencies from 20 kHz to several gigahertz. It has been applied in a wide range of fields including agriculture and forestry. Plant exposure to ultrasound also leads to production changes of secondary metabolites [50–52]. Here we provided a brief glance, as an addition to the role of ordinary sound in plants.

As a physical stress, ultrasound alone up-regulated resveratrol, taxol, ginsenoside saponins, shikonin, cartenoids, isoflavonoids and carotenoids. There also existed synergistic effects among ultrasound, ultraviolet irradiation, jasmonic acid and salicylic acid [50]. Further ingredient analysis showed that ultrasound or its combination with another physical or chemical stress may improve potential medicinal properties [51], such as enhancement of anti-diabetic properties of in vitro plant cell culture of Orthosiphon aristatus [52].

Even though the effect of sound on microorganism is not the key point in this review, numerous studies had shown that sound stimulated the growth and development of microorganisms [31, 47, 48], whether it was infrasonic, audible or ultrasonic vibration (Table 1).

**Table 1:** List of literature studies of secondary metabolite production in the presence of sound exposure

Region	Species	Sound Frequency	Exposure Time	Changes of Secondary Metabolites	Refs
China	taxus chinensis	50 Hz	30 min	Increased taxuyunnanine C and phenolics content	[10]
China	Chinese cabbage and cucumber	20 kHz; 75 dB	3 h / day	Increased polyamines and vitamin C levels in Chinese cabbage, as well as elevated polyamine levels in cucumber. “Green music” increased cucumber vitamin C content, sound waves decreased cucumber vitamin C content	[11]
China	chrysanthemum	1.4 kHz; 95 dB	1 h / day, twice a day, 20 days	Increased biosynthesis of 3-indoleacetic acid	[12]
Korea	alfalfa; broccoli; red young radish	250, 800, 1000, 1500 Hz; 80 dB	Short-term: twice daily for 1 h; Long-term: twice daily for 1 h, 2–4 days	Both short-term and long-term treatments increase total flavonoid content	[13]
Korea	tomato	1 kHz; 100 dB	6 h	Reduced ethylene production in tomato fruits	[14]
Korea	alfalfa	250, 500, 800, 1000, 1500 Hz; 80 dB	2 h / day, twice a day	Increased L-ascorbic acid content of alfalfa sprouts	[15]
Iran	saffron corms	0.5, 1, 2, 4, 8, 12, 16 kHz; 77 dB	15 min / day, 1–4 mouths	Crocin, picrocrocin, and safranal were lowest at 1 kHz and highest at 2 kHz. During the flowering stage, high frequencies increased the content of crocin, picrocrocin, and safranal	[16]
Iran	savory	Iranian music: 800–2000 Hz, 80 dB; rock music: 1100–7000 Hz, 80 dB; urban/traffic noise: 800–2000 Hz, 80 dB	three times a day for 1 h, two weeks	Increased total phenolic and soluble phenol concentrations. Urban/traffic noise treatment increased total flavonoid concentrations and the other treatments decreased total flavonoid concentrations	[7]
Turkey	strawberry plants	1000 Hz; 95, 100, 105 dB	1 h / day, 30 days	Total sugar content was highest at 100 dB, while total phenolic content and ascorbic acid increased with sound intensity	[17]

## Conclusion

To date, studies on sound’s function toward plants are still rare, and previous studies had some limitations. For example, sound and music had been known both beneficial and harmful for animals [53], but their effects on plants haven’t been clarified. Besides frequency and amplitude, sound and direction of sound are two important properties, which were neglected in literature. Sound may be processed into a plant cell via either of three types of media, air, liquid and solid. Comparison of sound stimulus among these three media was missing. On other hand, sound in nature is a mixture of a wide range of frequency, amplitude and pitch, which may be rhythmic, non-rhythmic, harmonic or non-harmonic. In order to understand plants’ perception, they need to be exposed to a wide diversity of sound: a single-frequency stimulus, a combination of stimuli, rhythmic and harmonic music, and non-rhythmic and non-harmonic noise.

On the other hand, plants have existed on Earth for millions of years with occupation on every ecological niche. What does a plant hear and know? In order to understand how plants hear and respond, we may also need to unravel the emission sound from plants, i.e. what plants talk about. This way, we can better design experiments of sound stimulation for secondary metabolites.

In short, the role of sound in production of plant secondary metabolites were summarized from molecular-, biochemical- and physiological levels. However, we are still at the infancy stage to understand plant acoustics, especially the effect of sound on secondary metabolites. From an applicative point of view, in-depth knowledge of plant’s sensitivity towards sound and music can be proficiently harnessed to stimulate the biosynthesis of targeted secondary metabolites, therefore obtaining bioactive enriched products with less hazardous chemical inputs, which can enhance sustainable natural environment.

## Abbreviations

Hz: Hertz
dB: Decibel
RIN: Ripening-inhibitor
ACS: ACC synthase
ACO: ACC oxidase
qRT-PCR: Quantitative real-time PCR
ACC: 1-Aminocyclopropane-1-carboxylic acid
PAL: Phenylalanine ammonia-lyase
C4H: Cinnamic acid 4-hydroxylase
4CL: 4-Coumaric acid: CoA ligase
CHS: Chalcone synthase
CHI: Chalcone isomerase
F3H: Flavanone 3-hydroxylase
F3′H: Flavonoid 3′-hydroxylase
FLS: Flavonol synthase
DFR: Dihydroflavonol 4-reductase
ANS: Anthocyanidin synthase
ANR: Anthocyanidin reductase
ChIP: Chromatin immunoprecipitation
PEF: Pulsed electric field
ROS: Reactive oxygen species
IAA: Indole acetic acid
ABA: Abscisic acid
